# Mean-field theory of the general-spin Ising model

**DOI:** 10.1140/epjb/s10051-025-01060-8

**Published:** 2025-10-15

**Authors:** Lourens Waldorp, Tuan Pham, Han L. J. van der Maas

**Affiliations:** University of Amsterdam, Nieuwe Achtergracht 129-B, 1018 NP Amsterdam, The Netherlands

## Abstract

**Abstract:**

Motivated by modelling in physics and other disciplines, such as sociology and psychology, we derive the mean field of the general-spin Ising model from the variational principle of the Gibbs free energy. The general-spin Ising model has $2k+1$ spin values, generated by $-(k-j)/k$, with $j=0,1,2\ldots ,2k$, such that for $k=1$ we obtain $-1,0,1$, for example; the Hamiltonian is identical to that of the standard Ising model. The general-spin Ising model exhibits spontaneous magnetisation, similar to the standard Ising model, but with the location translated by a factor depending on the number of categories $2k+1$. We also show how the accuracy of the mean field depends on both the number of nodes and node degree, and that the hysteresis effect decreases and saturates with the number of categories $2k+1$. Monte Carlo simulations confirm the theoretical results.

**Graphic abstract:**

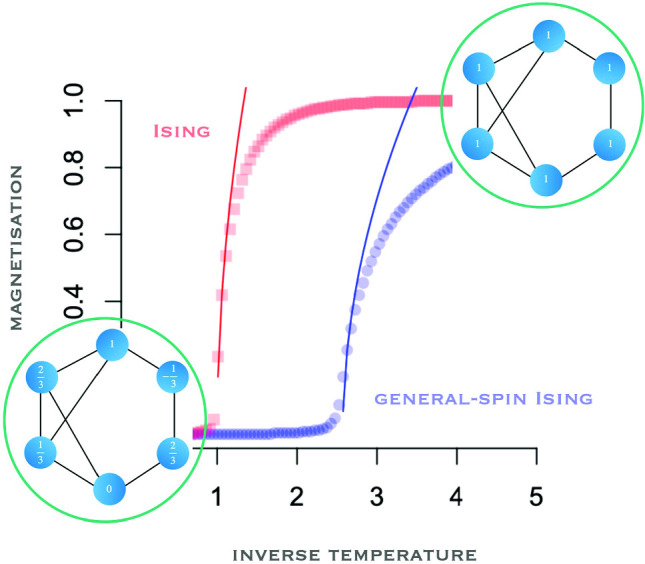

## Introduction

The Ising model is a popular model, used in physics but also in computational science, econophysics, sociophysics and psychosociophysics [6, 25, 28, 29, 42]. The model is relatively simple, yet exhibits intriguing phase transitions, both first- and second order. To obtain such qualitative descriptions, we explore mean-field theory of a generalisation of the Ising model that extends beyond the spin values $-1$ and 1 to include, for any $k\in \mathbb {N}$, the spin values generated by $(-k+j)/k$, with $j=0,1,2,\ldots ,2k$. For example, with $k=1$, we get $\{-1,0,1\}$. This model is called the general-spin Ising model and has been introduced before in Rabe and Mardia [34], where the interest was mostly in estimating parameters, and even earlier in Suzuki [39], which defined an Ising model for multiple states in general and derived the correlation functions.

On the face of it, the general-spin Ising model seems related to the continuous XY model and a discretisation of the Heisenberg model. However, the general-spin Ising model shows very different behaviour than both the standard Heisenberg and XY model; the general-spin Ising model has a first-order phase transition while the standard Heisenberg and XY model do not, even in the mean-field limit [3, 27]. The Blume-Capel model, which includes the value 0 and has an additional term for the energy of non-zeros (crystal field), has been investigated as a general-spin model [10, 32, 36]. The interest there was mostly in the effect of the crystal field term on magnetisation. Variations of the Ising model, such as the Potts model, are similar to the general-spin Ising model in that they involve multiple states; however, they only contribute to the energy when neighbouring states are equal. [17]; here any product of fractional values can contribute to the energy. The general-spin Ising model is different from the above models because it can be defined on the integers $\mathbb {Z}$, and here we normalise the values by the maximum *k*, so that all values are between -1 and 1.

Applications of the general-spin Ising model in physics mostly involve systems with quantized spin values [10] and mixed spin cases to model different magnetic materials [2, 15, 16]. For example, in Ertaş et al. [19] a two-layer square lattice is used, where nodes within each layer represent one of the materials in thin film, and interactions within and between layers are modelled according to the Ising model with spin values $\pm 2$, $\pm 1$, 0. However, our inspiration comes from the use of statistical physics models in sociology and psychology [41]. In sociophysics, for example, agents’ opinions on social networks (e.g., Facebook, Instagram) may span a range of ordered values, such as from extreme left to extreme right, including a neutral position [e.g. 9]. Similarly, in mathematical psychology, the Ising model is applied to phenomena such as attitudes [13] or major depression disorder [11], where node values can also vary along a spectrum, from negative to positive, again, including a neutral position. In both last two contexts, nodes are often measured by questionnaires, the most common of which are Likert scales. A typical Likert scale item asks respondents to rate their position on a 7-point scale ranging from ’strongly disagree’ to ’strongly agree’ [24]. Currently, visual analogue scale responses (sliders) are becoming popular [5, 23] which can also be covered by the general-spin Ising model with large *k*.

The Ising model is relevant to social sciences because it captures the interactions by the product of the variables. Known as the law of mass action [18], it can be found in research on human interactions, such as in opinion dynamics, e.g., the voter model [35, 38] and in epidemiology, e.g. in susceptible-infected-susceptible models [26], but also in research on intelligence [37], attitudes [14], and psychopathology [12, 40, 45].

Our interest is qualitative descriptions, and mean-field theory is appropriate for that purpose. Mean-field theory is correct in the thermodynamic limit (infinite graph size), but is still quite accurate when the mean degree of nearest neighbours is sufficiently high [21]. In physics this is often true, since the typical graph topology in physics is the *d*-dimensional lattice $\mathbb {Z}^d$ [e.g. 1, 20, 22]. Also in more modern applications of (variations of) the Ising model, versions of the *d*-dimensional lattice are used [19]. In social science applications, graph topologies often resemble a small world, and in neuroscience the topology often resembles a scale-free graph [4, 38], although sometimes the degree distribution resembles a Poisson distribution [9], suggesting an Erdös-Renyí random graph. In applications to psychopathology, the graph topology appears to be sparse and cannot be distinguished from an Erdös-Renyí random graph [8, 40].

We derive the mean-field equations for the general-spin Ising model with values between $-1$ and $+1$. After introducing the model and some notation in Sect. 2, we consider the mean field equations in Sect. 3. Then, in Section 4 we show that spontaneous magnetisation depends on an additional factor containing the number of categories $2k+1$. In Sect. 5 we show with Monte Carlo simulations that the results of the Metropolis algorithm for the general-spin Ising model correspond well to the theoretical predictions, including dependence on system size and the degree of nodes.

## General-spin Ising model

We have graph $\mathcal {G}$ consisting of *n* nodes (vertices) in the set $V=\{1,2,\ldots ,n\}$, and edges (connections) $E=\{(s,t):s,t\in V \, ;\, s \,\textrm{and} \, t\, \mathrm{are\, neighbours}\}$. The topology we use is a random (Erdös-Renyí) graph with probability of connecting $p_e$, independently and identically for each pair of nodes; this leads to on average $d=p_e(n-1)$ connections.

**Fig. 1:**
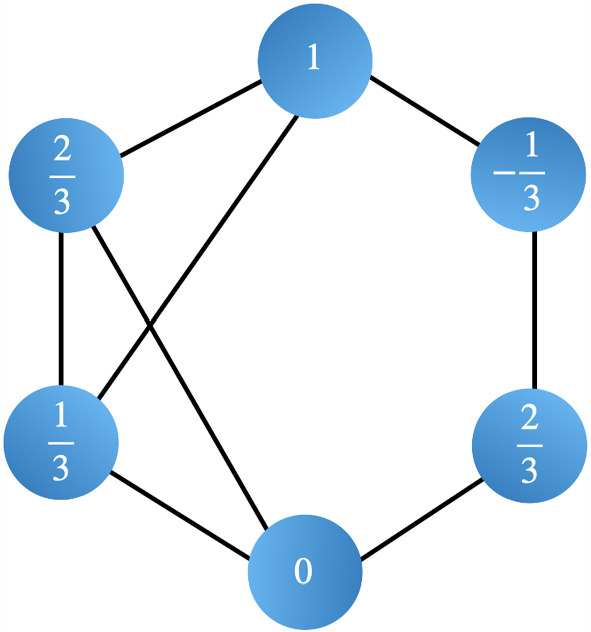
General-spin Ising model with $k=3$, and so there are 7 possible states

We assign to any node $s\in V$ a random variable $X_s$. The random variable $X_s$ can take values $x_s\in \Omega _k$ where $\Omega _k$ is generated by $(-k+j)/k$ for $j=0,1,2,\ldots ,2k$, that is,

$$\begin{aligned} \Omega _k:=\left\{ \frac{-k}{k},\frac{-k+1}{k},\frac{-k+2}{k},\ldots ,\frac{k-1}{k},\frac{k}{k}\right\} , \end{aligned}$$

for any finite natural number *k*. Note that the regular Ising model can be obtained by taking $k=\tfrac{1}{2}$, i.e. $\Omega _{1/2}=\{-1,1\}$. Figure 1 shows an example with $k=3$ and $\Omega _3=\{-1,-\tfrac{2}{3},-\tfrac{1}{3},0,\tfrac{1}{3},\tfrac{2}{3},1\}$.

Given external field parameter $\tau \in \mathbb {R}$ and (ferromagnetic) interaction parameter $\sigma \ge 0$, the Hamiltonian is

1 $$\begin{aligned} \mathcal {H}(x) = -\tau \sum _{s\in V} x_s - \sigma \sum _{(s,t)\in E} x_s x_t, \end{aligned}$$

where the sum over $(s,t)\in E$ runs over all edges. Throughout the paper, we shall refer to the general-spin Ising model with this Hamiltonian and $k\in \mathbb {N}$ as Ising(*k*). At a temperature *T*, the probability of finding any realisation $x=(x_1,x_2,\ldots ,x_n)$ of $X=(X_1,X_2,\ldots ,X_n)$ is a Boltzmann probability and is

2 $$\begin{aligned} \mathbb {P}_\theta (x) = \frac{1}{Z_\theta }\exp (-\beta \mathcal {H}(x)), \end{aligned}$$

where $\beta =1/T$ is the inverse temperature, $\theta =(\beta , \sigma ,\tau )$, and $Z_\theta$ is the partition function (normalising constant) $Z_\theta :=\sum _{\{x\}} \exp (-\beta \mathcal {H}(x))$, where $\sum _{\{x\}}$ denotes the summation over all $(2k+1)^n$ possible configurations.

**Fig. 2:**
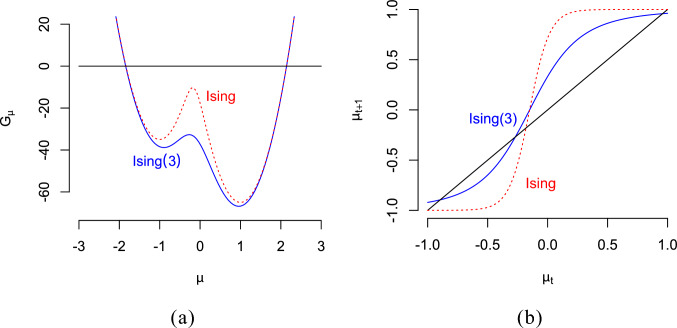
In (**a**) is the free energy function Eq. (3) with settings $\tau =0.3$, $d=2$, $\sigma =1$, $\beta =3$. For the general-spin Ising model *k* is 3 (blue) and the regular Ising model is shown for comparison (dashed, red). In (**b**) is an iterated map of the same general-spin Ising(3) model as in (**a**) with $\beta =3$, and the regular Ising model with the same settings

## Mean field

The mean field can be obtained by minimising the Gibbs free energy, which reveals the moments of the distribution [see e.g. 30, 44, Chapter3]. Assuming weak correlations between variables, we obtain the mean-field Hamiltonian (see Appendix A)

$$\begin{aligned} \mathcal {H}_{\mu }(x)=-\sigma \mu ^2 n \frac{d}{2} -(\sigma \mu d +\tau )\sum _{s\in V} x_s, \end{aligned}$$

where $\mu$ is the mean field, the average effect from neighbouring nodes, and *d* is the average degree of any node in the random graph $\mathcal {G}$. Using this mean-field Hamiltonian, the Gibbs free energy is

3 $$\begin{aligned} G_\mu&=-\frac{1}{\beta }\log Z_\mu = \sigma \mu ^2 n \frac{d}{2}\nonumber \\&\quad -\frac{n}{\beta }\log \sum _{x\in \Omega _k^+}2\cosh \left( \beta (\sigma \mu d+\tau )x\right) , \end{aligned}$$

where $\Omega _k^+=\{0,\tfrac{1}{k},\ldots ,1\}$. Minimising $G_\mu$ we obtain the mean field $\mu$ (Appendix A)

4 $$\begin{aligned} \mathbb {E}(x) =\mu&=\frac{\sum _{x\in \Omega _k^{+}}x\sinh (x\beta \gamma )}{\sum _{x\in \Omega _k^{+}}\cosh (x\beta \gamma )}, \end{aligned}$$

where $\gamma = \tau + \sigma \mu d$. The expression of $\mu$ for Ising(*k*) is similar in spirit to the one of the Ising model, except that we have a sum of terms ranging over $\Omega _k^+$. The susceptibility (variance) is obtained by taking the derivative of the mean field in (4) with respect to $\tau$ [33]. We obtain

5 $$\begin{aligned} \mathbb {E}(x^2)-(\mathbb {E}(x))^2=\chi = \beta \left( \frac{\sum _{x\in \Omega _k^{+}}x^2\cosh (x\beta \gamma )}{\sum _{x\in \Omega _k^{+}}\cosh (x\beta \gamma )}-\mu ^2\right) . \end{aligned}$$

Using the mean field we can investigate qualitatively some properties of the general-spin Ising model. Figure 2a shows the free energy of the mean field approach for the general-spin Ising model as well as for the regular Ising model. In this example, the general-spin Ising model has 7 spin values $-1,-\tfrac{2}{3},-\tfrac{1}{3},0,\tfrac{1}{3},\tfrac{2}{3},1$; we denote this version of the general-spin Ising model Ising(3), since $k=3$. From Fig. 2a three results are immediately clear. First, the number of local and global minima is the same for the Ising(3) as for the regular Ising model. Second, the perturbation necessary to switch to a lower minimum is smaller for the Ising(3) model than for the regular Ising model as the barrier’s height is lower. And third, the minima of the free energy of the general-spin Ising model appear closer to 0 than the minima of the regular Ising model.

**Fig. 3:**
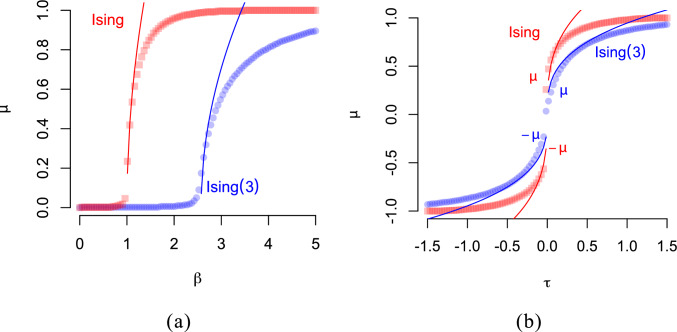
Magnetisation $\mu$ using the mean field with parameters $d=10$, $\sigma =0.1$, $\tau =0$ and $k=3$ for the general-spin Ising model (blue circles) and the regular Ising model (red squares). (**a**) is the magnetisation as a function of the inverse temperature $\beta$. In (**b**) is the magnetisation as a function of the external field $\tau$. The continuous lines are the predictions from equations (6) in (**a**) and (7) in (**b**)

In Fig. 2b we see an iteration map that can be used for a graphical analysis of the fixed points of the general-spin Ising model. On the *x*-axis we have the value of the mean field in Eq. (4) at time *t* and on the *y*-axis we see the value of the mean field at time $t+1$; we see a single iterate of the mean-field function. Both the general-spin Ising model with $k=3$ and the regular Ising model show three fixed points, where the middle fixed point is repelling and the two extreme fixed points are attracting. It also shows that the fixed points, corresponding to the minima of the free energy, are a bit closer to 0 for the general-spin Ising model than for the regular Ising model.

## Phase transitions

We first discuss the phase transition of the magnetisation $\mu$ as a function of the inverse temperature $\beta$, without an external field. Using Landau theory [e.g. 33], we obtain that the phase transition of the general-spin Ising model is similar to the (second-order) phase transition of the regular Ising model, but for the general-spin Ising model the location of spontaneous magnetisation is translated by a factor determined by the number of categories $2k+1$. In particular, the phase transition near spontaneous magnetisation $\mu =0$ is of order (see Appendix B)

6 $$\begin{aligned} \mu \approx {\left\{ \begin{array}{ll} \pm \sqrt{r\beta \sigma d-1} & \text { if } r\beta \sigma d \searrow 1\\ 0 & \text { if } r\beta \sigma d \le 1 \end{array}\right. }, \end{aligned}$$

where $r=(2k+1)/6k$ and *d* is the average degree of any node in the random graph $\mathcal {G}$. In terms of the critical temperature $\beta _c=1/r\sigma d$ we obtain $\mu \propto (\tfrac{\beta }{\beta _c}-1)^{1/2}$ if $\beta \searrow \beta _c$, and 0 otherwise. Interestingly, the factor *r* is between $\tfrac{1}{2}$ when $k=1$ (with categories $-1$, 0, and 1), and $r=\tfrac{1}{3}$ for $k\rightarrow \infty$. Near criticality, the susceptibility, is approximately $\chi \approx \beta (r-\mu ^2)$, for small $\mu$.

In Fig. 3a we show the magnetisation as a function of the inverse temperature, for both the general-spin Ising model with $k=3$ and the regular Ising model. While spontaneous magnetisation emerges in a similar way in both models, the critical value of the inverse temperature for the general-spin Ising model is shifted to the right by a factor of $r=(2k+1)/6k$.

The phase transition of the magnetisation $\mu$ as a function of the external field $\tau$ is obtained by considering its behaviour near the critical inverse temperature, which for the general-spin Ising model is $\beta _c=1/(r \sigma d)$. We obtain the following equation (Appendix B):

7 $$\begin{aligned} \mu \approx \text {sign}(\tau )\left( \frac{|\tau |}{r\sigma d}\right) ^{1/3}. \end{aligned}$$

This result can be leveraged to yield the decreasing width of the hysteresis. We expect a transition from $-1$ to $+1$ (or vice versa) to happen at $\tau \approx \pm r\sigma d$ (and equals $\pm 1/\beta _c$). And since *r* will be approximately $\tfrac{1}{3}$ for large values of *k*, we expect that the width of the hysteresis converges.

Figure 3b shows the phase transition for the general-spin Ising model and the regular Ising model as a function of the external field $\tau$. As before, the points represent spontaneous magnetisation, where there are two attractive fixed points at the extremes determined by the external field $\tau$. The lines represent the function of equation (7); without the factor *r* for the regular Ising model. We find that the mean field for the general-spin Ising model with $k=3$ undergoes a similar first-order phase transition but the magnetisation as a function of $\tau$, but does not exhibit a jump from one extreme to the other as large as what happens to the regular Ising model. The fact that the general-spin Ising model has multiple spin values makes this possible. Again, the factor $r=(2k+1)/6k$ plays a key role in determining spontaneous magnetisation.

**Fig. 4:**
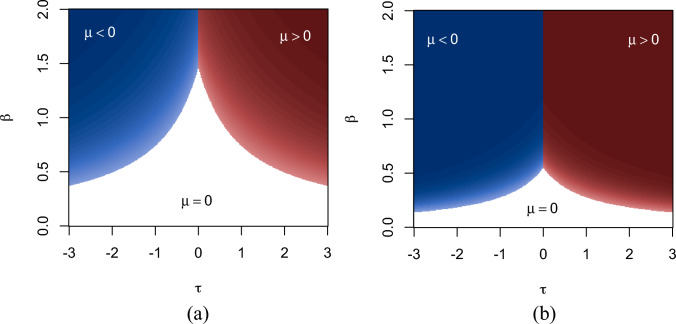
Phase maps for the Ising(3) model in (**a**) and the Ising model in (**b**). Shown in colours is the spontaneous magnetisation of the mean field as a function of the external field $\tau$ and inverse temperature $\beta$ from equation (4) with parameter settings $d=2$, $\sigma =1$, and $k=3$ in (**a**). Red colour indicates positive magnetisation, blue negative magnetisation and white 0 magnetisation

In Fig. 4a we show the phase map in the $\beta -\tau$-plane for the general-spin Ising model, and in (b)— that for the regular Ising model. For low values of the inverse temperature $\beta$, the spins are approximately random and so the magnetisation is $\mu =0$. For larger values of $\beta$ spontaneous magnetisation arises. Edges of the phase maps show the combination of $\beta$ and $\tau$ where spontaneous magnetisation arises, and remains occurring in the coloured areas; blue for negative magnetisation and red for positive magnetisation. Comparing the general-spin Ising model and the regular Ising model, the general-spin Ising model requires lower temperatures for spontaneous magnetisation than the regular Ising model.

Summarising, the mean-field behaviour of the general-spin Ising model shows very similar behaviour to the regular Ising model, but depends on an additional factor $r=(2k+1)/6k$, with $k\in \mathbb {N}$.

## Numerical illustration

To determine that the predictions from the mean field solution in (4) and its consequences for magnetisation in (6) and (7) are accurate, we perform Monte Carlo simulations. We use the single-site Metropolis algorithm [31, see Appendix C]. An Erdös-Rényi network is generated with varying probability of an edge $p_e$ and varying number of nodes *n*, so that the average degree *d* also varies. Connectivity is $\sigma =0.1$.

**Fig. 5:**
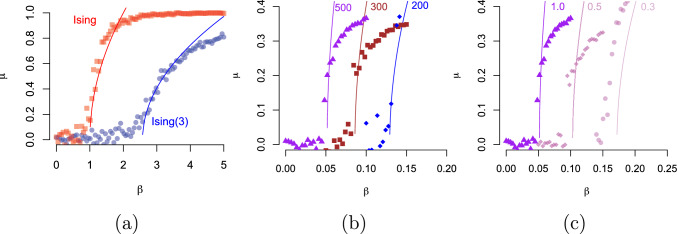
In (**a**) the magnetisation $\mu$ as a function of $\beta$ of the regular (red squares) and the general-spin Ising model with $k=3$ (blue circles) obtained with the Metropolis sampler and the theoretical values obtained with (6) as solid lines. The parameters are $n=100$, $\sigma =0.1$, $p_e=0.1$. In (**b**) is the magnetisation (6) a function of $\beta$ for different sizes of the complete graph with $n=200$, 300, and 500 nodes. In (**c**) is the magnetisation (6) as a function of $\beta$ for a random graph with $n=500$ nodes and edge probability $p_e=1$, 0.5, and 0.3

In Fig. 5a is the magnetisation $\mu$ as a function of $\beta$. The location of the spontaneous magnetisation of the general-spin Ising model (blue circles) confirms the theoretical predictions (solid line). Near criticality, we see in Fig. 5b that the accuracy of the mean field becomes worse with smaller graphs, and that this dependency also holds when the degree of each node is decreased [Fig. 5c; we left in $p_e=0.3$ to use for sparse graphs, as used in belief propagation on tree-like graphs, e.g., 7].

**Fig. 6:**
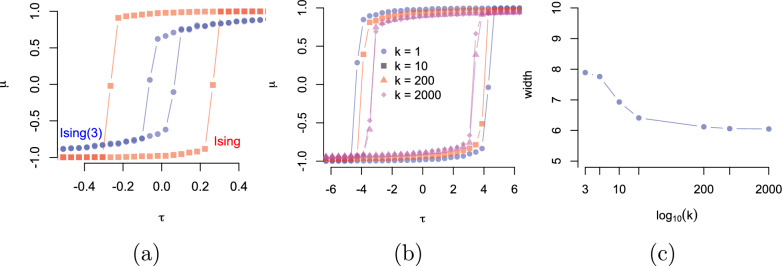
In (**a**) is the magnetisation as a function of the external field $\tau$ and with $\beta =5$. Increasing $\tau$ from $-0.5$ to 0.5 (circles), and going from 0.5 to $-0.5$ (squares). In (**b**) are hysteresis plots for the general-spin Ising model for different values of $k=1$, 10 and 200 and 2000. In (**c**) are the widths of the hysteresis for $k=3$ up to $k=2000$ in $\log$ with base 10. Other parameters are $\beta =1.1$, $n=100$, $p_e=0.1$

Figure 6a shows hysteresis of the magnetisation $\mu$ as a function of the external field $\tau$. This effect is because of the memory (residual) magnetisation when changing the external field. Comparing with the regular Ising model, the hysteresis effect is less strong (the upward and downward jumps are closer) for the general-spin Ising model than for the regular Ising model.

Figure 6b shows that there is a strong indication that the hysteresis effect converges to a particular point. It is easily proved that if each term $\sinh (x\beta \gamma )$ in the numerator and $\cosh (x\beta \gamma )$ in the denominator of the mean field expression in (4) is $<1$ (in absolute value), then with $k\rightarrow \infty$ the mean field will converge. This is sufficient because the partial series $\sum _{s=1}^k \tfrac{s}{k}$ has ratio test value 1. Figure 6c confirms this numerically.

## Conclusion and discussion

The general-spin Ising model, a model with multiple valued spins, symmetric around zero between $-1$ and $+1$, is important not only as a theoretical framework, but also because of its applications–ranging from *S*-spin models in physics to polarisation dynamics in sociology and attitude modelling in psychology. In such applications, it is often valuable to understand the types of states and dynamics the model can exhibit, to assess its suitability for a given modelling context. Mean-field theory offers insights into these properties.

We obtained the Gibbs free energy for the mean field and minimised this to obtain the mean field solution. The properties of the general-spin mean field are similar to those of the regular Ising model, i.e. the emergence of spontaneous magnetisation via a second-order transition at low temperatures, and hysteresis. However, spontaneous magnetisation occurs at a shifted value compared to the regular Ising model, determined by $r=(2k+1)/6k$, where $2k+1$ denotes the number of categories of the general-spin Ising model. The phase diagram showed that temperatures need to be lower to obtain spontaneous magnetisation in the general-spin Ising model than for the regular Ising model. Additionally, the hysteresis effect is slightly different depending on the number of categories, but converges when taking the limit of $k\rightarrow \infty$. Monte Carlo simulations confirm the theoretical predictions derived from the mean field, and indicate the dependence on the system size and node degree.

Using these results allows for the application of the general-spin Ising model in different fields. In psychology, for example, this model is applicable in contexts where responses are measured using a visual analogue scale, typically implemented as a slider with around 100 response categories. These types of responses could then be modelled with a general-spin Ising model with many categories. Experiments should reveal whether the predictions from mean-field theory accurately reflect empirical data.

## Figures and Tables

**Algorithm 1:**
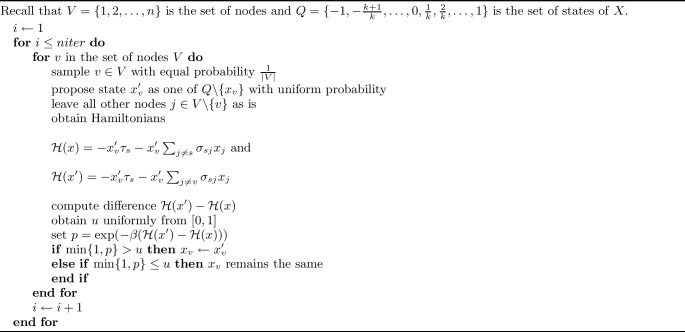
Metropolis algorithm

## Data Availability

This manuscript does not contain data.

## A Mean field derivation

Intuitively, the mean field assumes that in a well enough connected graph $\mathcal {G}$, the links are weak [see 21], such that we can obtain the measure $\mathbb {P}_\theta (x)$ in Eq. (2) as a product of the marginal distributions $\mathbb {P}^{(i)}_\theta (x_i)$, and so that the partition function is a sum of products [21, 33]. To construct the mean field we need two assumptions. These assumptions are similar to those described by, e.g., Plischke and Bergersen [33].

### Assumption 1

*(Large degree)* Each node in the graph $\mathcal {G}$ has sufficiently high degree *d*.

In practice we use a random (Erdös-Renyí) graph with sufficiently high probability of connection $p_e$, so that approximately $d=p_e(n-1)$ for each node.

### Assumption 2

*(Concentration)* The nodes (variables) $X_s$ in graph $\mathcal {G}$ are sub-gaussian random variables with mean $\mu$ and variance proxy $\sigma ^2$.

Assumption 2 is another (quantitative) way to ensure that the correlations between nodes remains low (see below); it implies that for any $s\in V$ we obtain by Hoeffding’s bound [43]

$$\begin{aligned} \mathbb {P}(|X_s-\mu |\ge \delta n)\le 2\exp \left( -\frac{\delta ^2 n^2}{\sigma ^2} \right) , \end{aligned}$$

where *n* is the number of nodes in $\mathcal {G}$, i.e. $n=|V|$. This concentration of measure is required for the mean field approximation, to keep the correlations between the variables small. Because the random variables $X_s$ are bounded between $-1$ and $+1$, we immediately obtain that they are sub-gaussian with variance proxy 1 [43].

To obtain the mean field we obtain the free energy and use the first-order derivative to obtain the first moment [e.g., 30]. We have the partition function

$$\begin{aligned} Z=\sum _{x\in \Omega _k}\exp \left( \beta \tau \sum _{s\in V} + \beta \sigma \sum _{(s,t)\in E} x_s x_t\right) . \end{aligned}$$

The interactions for random variables $X_s$ can be rewritten as

$$\begin{aligned}&X_s X_t=(X_s-\mu +\mu )(X_t-\mu +\mu )\\&\quad =(X_s-\mu )(X_t-\mu )+2\mu (X_s-\mu )+\mu ^2. \end{aligned}$$

By Assumption (2) the covariance term is small. Assuming that the random variables $X_s$ are sub-gaussian implies that

$$\begin{aligned} \mathbb {P}(|(X_s-\mu )(X_t-\mu )|\ge \delta n)\le 2 \exp \left( -\frac{n^2\delta ^2}{2\sigma ^2}+\log 2\right) . \end{aligned}$$

Therefore, taking the right hand side as $\epsilon$, so that $\delta =\sqrt{\tfrac{2\sigma ^2}{n^2}\log \tfrac{1}{\epsilon }}$, we obtain with high probability $1-\epsilon$ that

$$\begin{aligned} |(X_s-\mu )(X_t-\mu )|\le \sqrt{2\sigma ^2\log \frac{1}{\epsilon }}, \end{aligned}$$

and we see that the covariance terms are small. Relatedly, this result can be connected to the Ginzburg criterion [see e.g., 33], such that we demand that $\epsilon \ge \exp ( -\frac{\mu ^4}{2\sigma ^2})$, i.e. we demand that the mean field is large enough in the fourth order moment compared to the second order moment, i.e. the second-order fluctuations cannot be too large.

And so we have the mean field Hamiltonian

$$\begin{aligned} \mathcal {H}_{\mu }(x)=-\tau \sum _{s\in V} x_s-\sigma \sum _{(s,t)\in E}(-\mu ^2 +2\mu x_s). \end{aligned}$$

Noting that $\sum _{(s,t)}\mu ^2=\mu ^2 n d/2$, with *d* the degree of each node in $\mathcal {G}$ (by Assumption 1), and $\sum _{(s,t)} x_s= (d/2) \sum _s x_s$. We then obtain the mean field Hamiltonian

$$\begin{aligned} \mathcal {H}_{\mu }(x)=\sigma \mu ^2 n \frac{d}{2} -(\sigma \mu d +\tau )\sum _{s\in V} x_s. \end{aligned}$$

The partition function for the mean field $Z_\mu$ then becomes

$$\begin{aligned} Z_\mu&= \exp (-\beta \sigma \mu ^2 n d/2) \sum _{x_s\in \Omega _k}\exp \left( \beta (\sigma \mu d+\tau )\sum _{s\in V} x_s\right) , \end{aligned}$$

By Assumption 1 we see that we can replace each $x_s$ by a generic *x*. Hence, we obtain

$$\begin{aligned} Z_\mu&= \exp (-\beta \sigma \mu ^2 n d/2)\left( \sum _{x\in \Omega _k^+}2\cosh \left( \beta (\sigma \mu d+\tau )x\right) \right) ^n, \end{aligned}$$

where $\Omega _k^+=\{0,\tfrac{1}{k},\tfrac{2}{k},\ldots ,1\}$. We can then define the Gibbs free energy for the mean field as

$$\begin{aligned} G_\mu \!=\!-\frac{1}{\beta }\log Z_\mu \!=\! \sigma \mu ^2 n \frac{d}{2}\!-\! \frac{n}{\beta }\log \sum _{x\in \Omega _k^+}2\cosh \left( \beta (\sigma \mu d\!+\!\tau )x\right) , \end{aligned}$$

To obtain the mean field we need to minimise the free energy $G_\mu$, leading to

$$\begin{aligned} \mathbb {E}(x)&=\mu = \frac{\sum _{x\in \Omega _k^+}x\sinh (x\beta \gamma )}{\sum _{x\in \Omega _k^+}\cosh (x\beta \gamma )} \end{aligned}$$

where $$\gamma =\sigma \mu d +\tau $$.

## B Landau theory

We consider Landau theory for the mean field in (4). Initially, we set the external field $\tau =0$. The approximation is obtained by a Taylor series of (4) at $\mu =0$

$$\begin{aligned} \mu =a_0 + a_1\mu + a_2\mu ^2+a_3\mu ^3 +O(\mu ^4). \end{aligned}$$

The first coefficient is $a_0$, which can be seen to be 0 by setting in (4) $\mu =0$ in $\gamma$ and recalling that $\tau =0$. For $a_1$ we require the first derivative. Let $C=1+\cosh (\tfrac{1}{k}\beta \gamma )+\cosh (\tfrac{2}{k}\beta \gamma )+\cdots +\cosh (\beta \gamma )$. Then the derivative of (4) gives $a_1$

$$\begin{aligned} \beta \sigma d\frac{C\sum _{x\in \Omega _k^+}x^2\cosh (x\beta \gamma )}{C^2}&= \beta \sigma d\frac{(\frac{1}{k})^2+(\tfrac{2}{k})^2+\cdots +1}{k+1} \\&= \beta \sigma d\frac{2k+1}{6k}, \end{aligned}$$

where we already set $\mu =0$ so that all $\sinh$ terms are 0, and we used the fact that $\sum _{x\in \Omega _k^+}x^2=(k+1)(2k+1)/6k$. It is obvious that $a_2=0$ since all terms contain $\sinh$. Then, the next derivative gives

$$\begin{aligned}&a_3= (\beta \sigma d)^3\frac{C\sum _{x\in \Omega _k^+}x^4\cosh (x\beta \gamma )}{C^2}\\&\quad -3(\beta \sigma d)^3\frac{\left( \sum _{x\in \Omega _k^+}x^2\cosh (x\beta \gamma )\right) ^2}{C^2}, \end{aligned}$$

where we again already removed the $\sinh$ terms which are 0 at $\mu =0$. Then we obtain

$$\begin{aligned} a_3&= (\beta \sigma d)^3\frac{\sum _{x\in Q_+}x^4\cosh (x\beta \gamma )}{C^2} \\&\quad -3(\beta \sigma d)^3\frac{\left( \sum _{x\in Q_+}x^2\cosh (x\beta \gamma )\right) ^2}{C^2}. \end{aligned}$$

Using that $\sum _{x\in [n]} x^4=k(k+1)(2k+1)(3k^2+3k-1)/30k^4$, we get

B.1 $$\begin{aligned} a_3= (\beta \sigma d)^3\left( \frac{k(2k+1)(3k^2+3k-1)}{30(k+1)^3}-3\frac{r^2}{(k+1)^2}\right) , \end{aligned}$$

where again we have set $\mu$ to 0 so that $\sinh$ terms drop out and where $r=(2k+1)/6k$. This gives the approximation

$$\begin{aligned} \mu = r\beta \sigma d \mu - \frac{1}{3}a_3\mu ^3 + O(\mu ^5). \end{aligned}$$

And so, approximately

$$\begin{aligned} \mu \approx \pm a_3^{-1/2}\sqrt{3}\sqrt{r\beta \sigma d-1}. \end{aligned}$$

So, there is a second-order transition when $r\beta \sigma d$ approaches 1 from above, and if $r\beta \sigma d\le 1$, then the magnetisation is 0.

We can obtain a similar result when including the external field. In the above Taylor expansion we use $r\beta _c\sigma \mu d +\beta _c\tau$, where $\beta _c$ is the critical inverse temperature $(r\sigma d)^{-1}$, so that we have $\mu +\beta _c\tau$ [33, Section3.6]. Then we obtain

$$\begin{aligned} \mu = \mu + \beta _c\tau - \frac{1}{3}a_3(\mu +\beta _c\tau )^3 + O(\mu ^5). \end{aligned}$$

And we obtain that the magnetisation changes according to the external field approximately as

$$\begin{aligned} \mu \approx \text {sign}(\tau )\left( \frac{3|\tau |}{a_3 r\sigma d}\right) ^{1/3}. \end{aligned}$$

## Code availability

Pseudo code for the simulations is available in the appendix. Code for fitting these models is in progress and will be made available at a later data.

## References

[1] M. Aizenman, D.J. Barsky, R. Fernández, The phase transition in a general class of ising-type models is sharp. J. Stat. Phys. **47**(3–4), 343–374 (1987)

[2] E. Albayrak, A. Yigit, Mixed spin-3/2 and spin-5/2 ising system on the bethe lattice. Phys. Lett. A **353**(2–3), 121–129 (2006)

[3] M. Barma et al., Solution of the random field xy magnet on a fully connected graph. J. Phys. A: Math. Theor. **55**(9), 095001 (2022)

[4] D.S. Bassett, E. Bullmore, Small-world brain networks. Neuroscientist **12**(6), 512–523 (2006) 17079517 10.1177/1073858406293182

[5] A. Betella, P.F. Verschure, The affective slider: a digital self-assessment scale for the measurement of human emotions. PLoS ONE **11**(2), e0148037 (2016) 26849361 10.1371/journal.pone.0148037 PMC4743948

[6] C. Castellano, S. Fortunato, V. Loreto, Statistical physics of social dynamics. Rev. Mod. Phys. **81**(2), 591–646 (2009)

[7] A. Castro, T.M. Pham, E. Ortega, D. Machado, Xenophobia based on a few attributes can impede society’s cohesiveness (2025). arXiv preprint arXiv:2506.18513

[8] D. Castro, J. Cardoso, A.S. Araujo, A.R. Rodrigues, F. Ferreira, F. Ferreira-Santos, T.B. Ferreira, Topological properties of psychopathological networks of healthy and disordered individuals across mental disorders. J. Affect. Disord. **366**, 226–233 (2024) 39216639 10.1016/j.jad.2024.08.168

[9] Y.C. Chen, M. H. S. . H. J. W. Multi-state open opinion model based on positive and negative social influences. In Proceedings of the 2015 IEEE/ACM International Conference on Advances in Social Networks Analysis and Mining, pages 170–177. IEEE/ACM (2015)

[10] E. Costabile, J.R. Viana, J.R. de Sousa, J. Plascak, The general-spin blume-capel model: a study of the multicritical behavior using effective-field theory. XXPhys. A **393**, 297–303 (2014)

[11] A.O.J. Cramer, C.D. van Borkulo, E.J. Giltay, H.L.J. van der Maas, K.S. Kendler, M. Scheffer, D. Borsboom, Major depression as a complex dynamic system. PLoS ONE **11**, e0167490 (2016) 27930698 10.1371/journal.pone.0167490 PMC5145163

[12] A.O.J. Cramer, L.J. Waldorp, H.L.J. Maas, D. Borsboom, Comorbidity: a network perspective. Behav. Brain Sci. **33**(2–3), 137–150 (2010) 20584369 10.1017/S0140525X09991567

[13] J. Dalege, D. Borsboom, F. van Harreveld, H.L. van der Maas, The attitudinal entropy (ae) framework as a general theory of individual attitudes. Psychol. Inq. **29**(4), 175–193 (2018)

[14] J. Dalege, D. Borsboom, F. van Harreveld, L.J. Waldorp, H.L. van der Maas, Network structure explains the impact of attitudes on voting decisions. Sci. Rep. **7**(1), 1–11 (2017) 28687776 10.1038/s41598-017-05048-y PMC5501836

[15] N. De La Espriella, G. Buendia, J. Madera, Mixed spin-1 and spin-2 ising model: study of the ground states. J. Phys. Commun. 2(2) (2018)

[16] B. Deviren, M. Keskin, O. Canko, Kinetics of a mixed spin-1/2 and spin-3/2 ising ferrimagnetic model. J. Magn. Magn. Mater. **321**(5), 458–466 (2009)

[17] R. Durrett, *Random graph dynamics* (Cambridge University Press, 2007)

[18] P. Érdi, J. Tóth, *Mathematical models of chemical reactions: theory and applications of deterministic and stochastic models* (Manchester University Press, 1989)

[19] M. Ertaş, M. Batı, Ü. Temizer, Dynamic hysteresis loops of the spin-2 bilayer ising model. Chin. J. Phys. **56**(3), 807–818 (2018)

[20] H.-O. Georgii, O. Häggström, C. Maes, The random geometry of equilibrium phases. Phase Trans. Crit. Phenom. **18**, 1–142 (2001)

[21] J.P. Gleeson, S. Melnik, J.A. Ward, M.A. Porter, P.J. Mucha, Accuracy of mean-field theory for dynamics on real-world networks. Phys. Rev. E-Stat. Nonlinear Soft Matter Phys. **85**(2), 026106 (2012) 10.1103/PhysRevE.85.026106 22463278

[22] G. Grimmett, *Probability on graphs: Random processes on graphs and lattices* (Cambridge University Press, Cambridge, 2010)

[23] J. Haslbeck, A.J. Martínez, A.J. Roefs, E.I. Fried, L.H. Lemmens, E. Groot, P.A. Edelsbrunner, Comparing likert and visual analogue scales in ecological momentary assessment. Behav. Res. Methods **57**(8), 1–41 (2025) 10.3758/s13428-025-02706-2 PMC12222338 40603809

[24] A.T. Jebb, V. Ng, L. Tay, A review of key likert scale development advances: 1995–2019. Front. Psychol. **12**, 637547 (2021) 34017283 10.3389/fpsyg.2021.637547 PMC8129175

[25] M. Jusup, P. Holme, K. Kanazawa, M. Takayasu, I. Romić, Z. Wang, S. Geček, T. Lipić, B. Podobnik, L. Wang et al., Social physics. Phys. Rep. **948**, 1–148 (2022)

[26] M. Keeling, K. Eames, Networks and epidemic models. J. R. Soc. Interface **2**, 295–307 (2005) 16849187 10.1098/rsif.2005.0051 PMC1578276

[27] K. Kirkpatrick, E. Meckes, Asymptotics of the mean-field heisenberg model. J. Stat. Phys. **152**(1), 54–92 (2013)

[28] K. Lee, S. Chowdhury, K.Y. Camsari, Noise-augmented chaotic ising machines for combinatorial optimization and sampling. Commun. Phys. **8**(1), 35 (2025)

[29] M.W. Macy, B.K. Szymanski, J.A. Hołyst, The ising model celebrates a century of interdisciplinary contributions. NPJ Complexity **1**(1), 10 (2024)

[30] R.E. McCulloch, Information and the likelihood function in exponential families. Am. Stat. **42**(1), 73–75 (1988)

[31] N. Metropolis, A.W. Rosenbluth, M.N. Rosenbluth, A.H. Teller, E. Teller, Equation of state calculations by fast computing machines. J. Chem. Phys. **21**(6), 1087–1092 (1953) 10.1063/5.0309018 41342507

[32] J. Plascak, J. Moreira et al., Mean field solution of the general spin blume-capel model. Phys. Lett. A **173**(4–5), 360–364 (1993)

[33] M. Plischke, B. Bergersen, Equilibrium statistical physics. World Scientific Publishing Company, 3rd edition (2006)

[34] S. Rabe, K. Mardia, Generalized ising models and their applications. J. Appl. Stat. **21**(5), 479–494 (1994)

[35] S. Redner, Reality-inspired voter models: a mini-review. C R Phys. **20**(4), 275–292 (2019)

[36] M. Salama, E. Choubabi, N. Hachem, K. Kihel, T. Mouhrach, M. Bouziani, Magnetic behavior of spin-3/2 blume-capel graphene-like monolayer in a transverse crystal field. Eur. Phys. J. B **97**(3), 25 (2024)

[37] A.O. Savi, M. Marsman, H.L. van der Maas, G.K. Maris, The wiring of intelligence. Perspect. Psychol. Sci. **14**(6), 1034–1061 (2019) 31647746 10.1177/1745691619866447 PMC7433699

[38] P. Sen, B.K. Chakrabarti, Sociophysics: an introduction. OUP Oxford (2014)

[39] M. Suzuki, Generalized exact formula for the correlations of the ising model and other classical systems. Phys. Lett. **19**(4), 267–268 (1965)

[40] C. van Borkulo, L. Boschloo, D. Borsboom, B.W. Penninx, L.J. Waldorp, R.A. Schoevers, Association of symptom network structure with the course of longitudinal depression. JAMA Psychiatry, pages 1219–1226 (2015) 10.1001/jamapsychiatry.2015.2079 26561400

[41] H. Maas, *Complex systems research in psychology* (Santa Fé Publisher, Santa Fé, 2024)

[42] H.L. Maas, J. Dalege, L. Waldorp, The polarization within and across individuals: the hierarchical ising opinion model. J. Compl. Netw. **8**(2), 10 (2020)

[43] R. Vershynin, *High-dimensional probability: An introduction with applications in data science,* vol. 47 (Cambridge University Press, 2018)

[44] M.J. Wainwright, M.I. Jordan, Graphical models, exponential families, and variational inference. Found. Trends Mach. Learn. **1**(1–2), 1–305 (2008)

[45] L. Waldorp, M. Marsman, G. Maris, Logistic regression and ising networks: prediction and estimation when violating lasso assumptions. Behaviormetrika **46**(1), 49–72 (2019)

